# Liver X receptors regulate natural killer T cell population and antitumor activity in the liver of mice

**DOI:** 10.1038/s41598-021-02062-z

**Published:** 2021-11-19

**Authors:** Kaori Endo-Umeda, Hiroyuki Nakashima, Shigeyuki Uno, Shota Toyoshima, Naoki Umeda, Shihoko Komine-Aizawa, Shuhji Seki, Makoto Makishima

**Affiliations:** 1 Division of Biochemistry, Department of Biomedical Sciences, Nihon University School of Medicine, Itabashi-ku, Tokyo Japan; 2 Department of Immunology and Microbiology, National Defense Medical College, Tokorozawa, Saitama Japan; 3 Allergy and Immunology Research Project Team, Research Institute of Medical Science, Center for Institutional Research and Medical Education, Nihon University School of Medicine, Itabashi-ku, Tokyo Japan; 4 Division of Microbiology, Department of Pathology and Microbiology, Nihon University School of Medicine, Itabashi-ku, Tokyo Japan; 5 Department of Biochemistry and Molecular Biology, Nippon Medical School, Bunkyo-ku, Tokyo Japan

**Keywords:** Tumour immunology, Molecular biology

## Abstract

The nuclear receptors liver X receptor α (LXRα) and LXRβ are lipid sensors that regulate lipid metabolism and immunity. Natural killer T (NKT) cells, a T cell subset expressing surface markers of both natural killer cells and T lymphocytes and involved in antitumor immunity, are another abundant immune cell type in the liver. The potential function of the metabolic regulators LXRα/β in hepatic NKT cells remains unknown. In this study, we examined the role of LXRα and LXRβ in NKT cells using mice deficient for LXRα and/or LXRβ, and found that hepatic invariant NKT (iNKT) cells are drastically decreased in LXRα/β-KO mice. Cytokine production stimulated by the iNKT cell activator α-galactosylceramide was impaired in LXRα/β-KO hepatic mononuclear cells and in LXRα/β-KO mice. iNKT cell-mediated antitumor effect was also disturbed in LXRα/β-KO mice. LXRα/β-KO mice transplanted with wild-type bone marrow showed decreased iNKT cells in the liver and spleen. The thymus of LXRα/β-KO mice showed a decreased population of iNKT cells. In conclusion, LXRα and LXRβ are essential for NKT cell-mediated immunity, such as cytokine production and hepatic antitumor activity, and are involved in NKT cell development in immune tissues, such as the thymus.

## Introduction

The liver is home to a variety of immune cells, including resident Kupffer cells, bone marrow-derived macrophages (BMDMs), natural killer (NK) cells, natural killer T (NKT) cells, T lymphocytes and B lymphocytes^1^. An understanding of the roles of hepatic immune cells is important to develop therapeutic and preventative strategies for liver diseases, such as nonalcoholic steatohepatitis and hepatocellular carcinoma^2^. NKT cells are a subset of T lymphocytes expressing surface markers common to NK cells and T lymphocytes, and recognize lipid antigens, such as α-galactosylceramide (α-GalCer), which are presented with the major histocompatibility complex class I-like molecule CD1d on antigen-presenting cells^3^. Invariant NKT (iNKT) cells, which express a semi-invariant T cell receptor (TCR) containing Vα14-Jα18 and Vβ11 in human and Vα14-Jα18 and Vβ8.2, Vβ7 or Vβ2 in mice, are enriched in mouse liver (20–30% of hepatic lymphocytes) and possess a strong activity for the production of the Th1 cytokine interferon-γ (IFN-γ) and the Th2 cytokine interleukin-4 (IL-4)^4^. Hepatic iNKT cells are involved in inflammation, antitumor immunity, repair and regeneration in mice^5–8^. Impaired iNKT cell function is associated with several human cancers, including hepatocellular carcinoma^9^, and NKT cell therapy is a promising approach for antitumor immunotherapy, such as chimeric antigen receptor NKT cell therapy against neuroblastoma^10,11^. Although iNKT cells play an important role in hepatic immunity, the function of metabolism regulators in iNKT cells remains largely unknown.

The nuclear receptors liver X receptor α (LXRα) and LXRβ are transcription factors that are activated by oxysterols and regulate expression of genes involved in lipid metabolism^12^. LXRα is abundantly expressed in liver, intestine, adipose tissue, kidney and macrophages, while LXRβ is ubiquitously present in the body. LXRs also regulate immune responses in immune cells, such as macrophages, through transrepression of other transcription factors, including nuclear factor κB and activation factor-1, and induction of genes involved in lipid metabolism, including the cholesterol efflux transporter ATP-binding cassette (ABC) transporter A1 (ABCA1)^13–16^. Recently, we found that LXRα and LXRβ are expressed in hepatic mononuclear cells (MNCs), including F4/80^+^ Kupffer cells/macrophages^17^. LXRα/β-knockout (KO) mice show increased numbers of F4/80^lo^CD11b^+^ Kupffer cells/macrophages in the liver and enhanced inflammatory responses after treatment with lipopolysaccharide or CpG-DNA. In addition, high-fat and high-cholesterol diet feeding leads to the development of severe nonalcoholic steatohepatitis in LXRα-KO mice, associated with an increase in F4/80^+^CD68^+^CD11b^+^ Kupffer cells/macrophages^18^. Interestingly, high-fat and high-cholesterol diet-fed LXRα-KO mice have a decreased proportion of iNKT cells in the liver and are resistant to inflammatory responses to α-GalCer or concanavalin-A (Con-A) treatment. Thus, LXRα and LXRβ regulate immune responses mediated by iNKT cells in addition to Kupffer cells/macrophages in the liver. In this study, we investigated the role of LXRα and LXRβ in hepatic iNKT cell-mediated immunity using LXRα-KO, LXRβ-KO and LXRα/β-KO mice and found that both LXRs are necessary for iNKT cell development and related immune responses.

## Results

### Decreased population of iNKT cells in the liver and spleen of LXRα/β-KO mice

**Figure 1:**
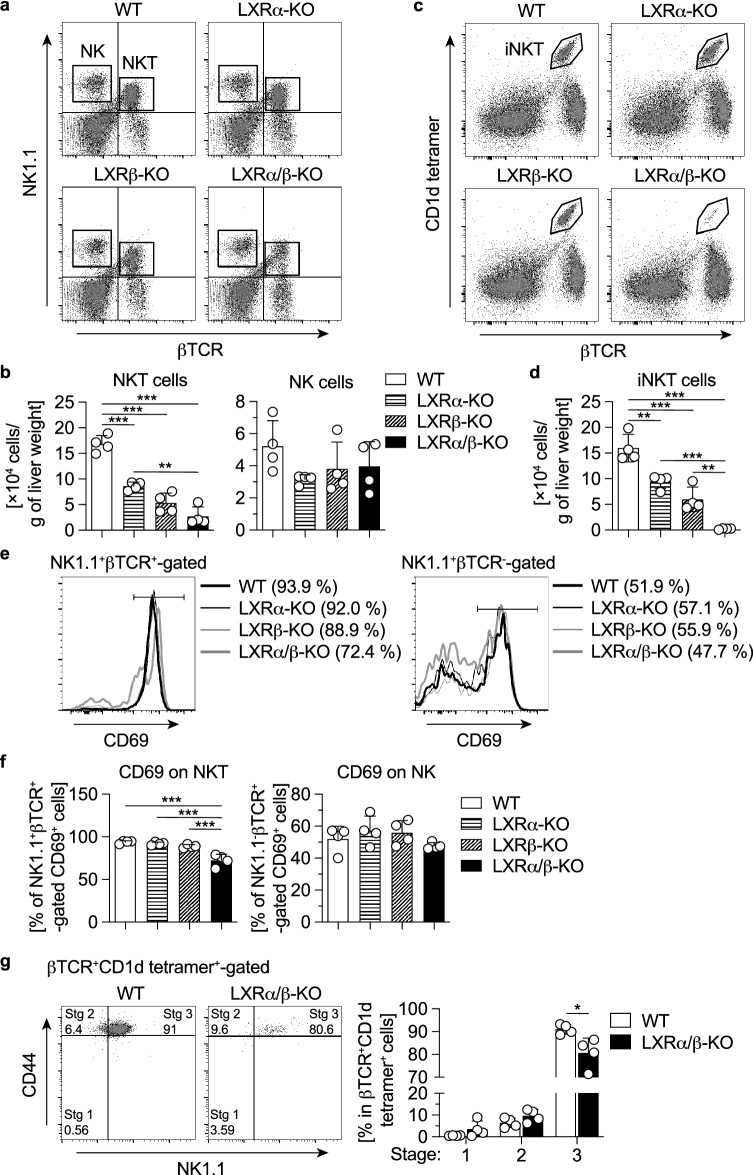
Decreased population of iNKT cells in the liver of LXRα/β-KO mice. (**a**) Representative flow cytometry for NKT cells (NK1.1^+^βTCR^+^) and NK cells (NK1.1^+^βTCR^−^) in hepatic MNCs. (**b**) Numbers of NKT cells and NK cells. (**c**) Representative flow cytometry for iNKT cells (βTCR^+^CD1d-tetramer^+^). (**d**) Numbers of iNKT cells. (**e**) Representative flow cytometry for CD69 expression in NK1.1^+^βTCR^+^-gated NKT cells and NK1.1^+^βTCR^−^-gated NK cells. (**d**) Numbers of CD69-positive cells in NKT cells and NK cells. (**f**) Percentages of NK1.1^−^CD44^−^ cells (stage 1), NK1.1^−^CD44^+^ cells (stage 2) and NK1.1^+^CD44^+^ cells (stage 3) in iNKT cells. Hepatic MNCs were isolated from WT, LXRα-KO, LXRβ-KO and LXRα/β-KO mice and stained with fluorescein isothiocyanate-conjugated (FITC-conjugated) anti-βTCR, phycoerythrin-conjugated (PE-conjugated) anti-NK1.1, biotin-conjugated anti-CD69 and PE-Cy5-streptavidin (**a**, **b**, **d** and **e**), with PE-conjugated CD1d tetramer and PE-Cy5-conjugated anti-βTCR (**c** and **d**) or with FITC-conjugated anti-NK1.1, PE-conjugated CD1d tetramer, PE-Cy5-conjugated anti-βTCR and allophycocyanin-conjugated (APC-conjugated) anti-CD44 (**g**) (n = 4). (**a**-**f**) **P < 0.01, ***P < 0.001 (one-way ANOVA followed by Tukey’s multiple comparisons). (**g**) Stg, stage. *P < 0.05 (Student’s *t* test).

We previously reported that the total number of MNCs and a population of F4/80^lo^CD11b^+^ BMDMs are increased in the liver of LXRα/β-KO mice^17^. We examined the effect of deletion of LXRα and LXRβ on the population of other types of hepatic immune cells. The number of hepatic NKT cells (βTCR^+^NK1.1^+^) was decreased in LXRα-KO mice and LXRβ-KO mice compared to wild-type (WT) mice, and further lowered in LXRα/β-KO mice (Fig. 1a, b). In contrast, the number of NK cells (βTCR^−^NK1.1^+^) was not affected by deletion of LXRα or LXRβ. Interestingly, iNKT cells (βTCR^+^CD1d-tetramer^+^) were drastically decreased in the liver of LXRα/β-KO mice, while the cell number was decreased to half and one-third of WT mice in LXRα-KO mice and LXRβ-KO mice, respectively (Fig. 1c, d). The percentage of NKT cells positive for CD69, an activation marker in NK cells and NKT cells^19^, was decreased in the liver of LXRα/β-KO mice, while CD69^+^ cells were not altered in NK cells in all groups of mice (Fig. 1e, f). Since NKT cells are matured in thymus and peripheral organs such as liver^3,20^, we examined the surface expression of CD44 and NK1.1 in βTCR^+^CD1d-tetramer^+^ cells from the liver. iNKT cells in stage 1 (CD44^−^NK1.1^−^) and stage 2 (CD44^+^NK1.1^−^) were comparable between WT and LXRα/β-KO mice, whereas stage 3 (CD44^+^NK1.1^+^) cells were significantly decreased in LXRα/β-KO mice. (Fig. 1g).Thus, NKT cells, particularly iNKT cells, are decreased in the liver of LXRα-KO mice and LXRβ-KO mice, with more severe deficiency in the liver of LXRα/β-KO mice.

Next, we examined NKT cell abundance in the spleen of LXR-deficient mice. Similar to the findings in the liver, splenic NKT cells were decreased in LXRα/β-KO mice, but not in LXRα-KO mice or LXRβ-KO mice (Supplementary Fig. 1a, b). iNKT cells were significantly decreased in the spleen of LXRα/β-KO mice (Supplementary Fig. 1c, d). The number of NK cells was not significantly altered in LXRα-KO, LXRβ-KO or LXRα/β-KO mice compared to WT mice (Supplementary Fig. 1b). There was no difference in the percentage of CD69^+^ NKT cells or NK cells in all groups of mice (Supplementary Fig. 1e, f). iNKT cells in stage 1 were increased and those in stage 3 were decreased in LXRα/β-KO mice compared to WT mice (Supplementary Fig. 1g). Therefore, LXRα/β deletion decreases NKT cells, particularly iNKT cells, in the liver and spleen.

### LXRα/β-KO hepatic iNKT cells are incompetent in cytokine production

**Figure 2:**
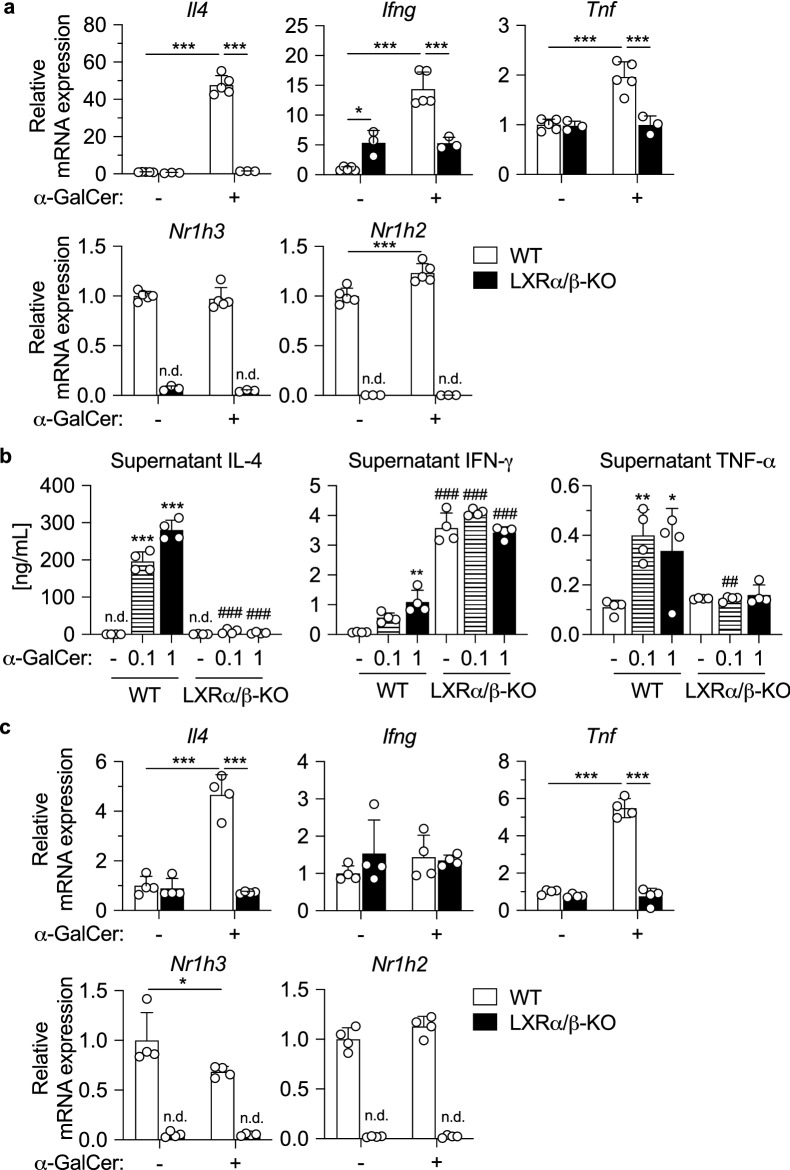
Impaired cytokine production in NKT cells from the liver and spleen of LXRα/β-KO mice. (**a**) mRNA expression of *Il4*, *Ifng*, *Tnf*, *Nr1h3* and *Nr1h2* in hepatic MNCs activated by the iNKT cell activator α-GalCer. Hepatic MNCs were isolated form WT and LXRα/β-KO mice and cultured with vehicle control (dimethyl sulfoxide) or α-GalCer (10 ng/ml) for 6 h (n = 3–5). **P < 0.01, ***P < 0.001 (one-way ANOVA followed by Tukey’s multiple comparisons). (**b**) Protein levels of IL-4, IFN-γ and TNF-α in culture media of hepatic MNCs simulated with α-GalCer. Hepatic MNCs were cultured with vehicle control (dimethyl sulfoxide), 0.1 or 1 µg/ml α-GalCer for 16 h, and concentrations of IL-4, IFN-γ and TNF-α in culture media were measured with enzyme-linked immunosorbent assay (n = 4). n.d., not detected. *P < 0.05, **P < 0.01, ***P < 0.001 compared to vehicle control treatment (one-way ANOVA followed by Tukey’s multiple comparisons). ##*P* < 0.01, ###*P* < 0.001 compared to WT cells with the same treatment (Student’s *t* test). (**c**) mRNA expression of *Il4*, *Ifng*, *Tnf*, *Nr1h3* and *Nr1h2* in splenocytes stimulated with α-GalCer. Splenocytes were isolated from WT and LXRα/β-KO mice and cultured with vehicle control (dimethyl sulfoxide) or α-GalCer (1 ng/ml) for 6 h (n = 4). n.d., not detected. *P < 0.05, ***P < 0.001 (one-way ANOVA followed by Tukey’s multiple comparisons).

To examine the effect of LXR deletion on NKT cell cytokine production in vitro, we isolated hepatic MNCs from WT and LXRα/β-KO mice, cultured with the iNKT cell-specific activator α-GalCer, and analyzed for expression of cytokine genes. In WT cells, α-GalCer treatment effectively induced mRNA expression of *Il4* (the gene encoding IL-4), *Ifng* (the gene encoding IFN-γ) and *Tnf* (the gene encoding tumor necrosis factor-α (TNF-α)) (Fig. 2a). In contrast, expression of these genes was not altered after α-GalCer treatment in LXRα/β-KO cells, and *Ifng* mRNA levels in untreated LXRα/β-KO cells were higher than in WT cells. Expression of *Nr1h3* (the gene encoding LXRα) was unaffected and that of *Nr1h2* (the gene encoding LXRβ) was slightly increased by α-GalCer treatment in WT cells. Interestingly, secreted IFN-γ protein levels were much higher in untreated LXRα/β-KO hepatic MNCs than in WT cells (Fig. 2b), consistent with *Ifng* mRNA expression (Fig. 2a). α-GalCer stimulation increased protein levels of IL-4, IFN-γ and TNF-α in conditioned media of WT cells but not in LXRα/β-KO cells (Fig. 2b). Impaired *Il4* and *Tnf* mRNA induction was also observed in splenocytes from LXRα/β-KO mice, while WT cells increased expression of these genes efficiently after α-GalCer treatment (Fig. 2c). *Ifng* mRNA expression was not changed both in WT and LXRα/β-KO splenocytes at 6 h after α-GalCer stimulation. These findings indicate that iNKT cell responses are impaired in hepatic MNCs and splenocytes derived from LXRα/β-KO mice.

**Figure 3:**
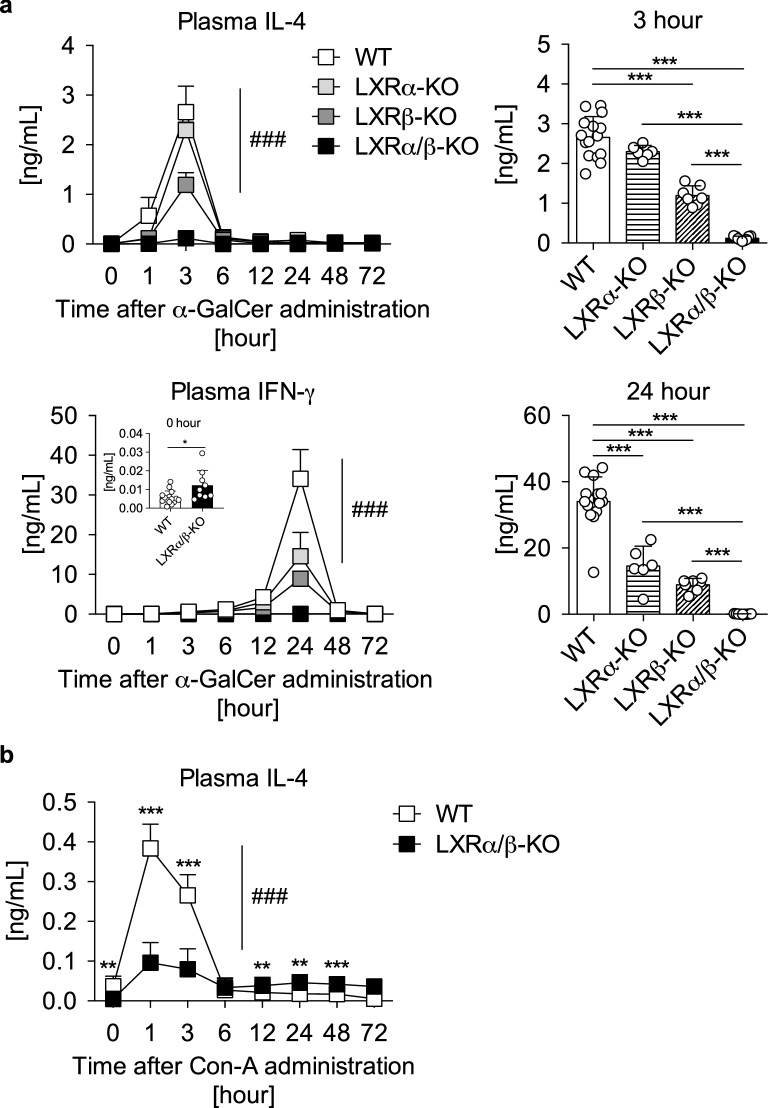
NKT cell-dependent cytokine production is impaired in LXRα/β-KO mice. (**a**) Plasma IL-4 and IFN-γ levels in mice treated with α-GalCer. WT (n = 15), LXRα-KO (n = 15), LXRβ-KO (n = 7) and LXRα/β-KO mice (n = 9) were injected with α-GalCer (0.1 mg/kg of body weight) intravenously, and plasma concentrations of IL-4 and IFN-γ were measured at the indicated time points. An inset panel in plasma IFN-γ shows the concentrations at the 0 h (unstimulated) time point. IL-4 levels at 3-h time point and IFN-γ levels at 24-h time point are also shown in bar graphs. ###*P* < 0.001 (two-way ANOVA). ***P < 0.001 (one-way ANOVA followed by Tukey’s multiple comparisons). (**b**) Plasma IL-4 levels in mice treated with Con-A. WT (n = 13) and LXRα/β-KO mice (n = 7) were treated with Con-A (12.5 mg/kg of body weight) intravenously, and plasma IL-4 concentrations were measured at the indicated time points. ###*P* < 0.001 (two-way ANOVA). **P < 0.01, ***P < 0.001 compared to WT at the same time point (Student’s *t* test).

We next examined iNKT cell responses in vivo by injecting α-GalCer intravenously to WT, LXRα-KO, LXRβ-KO and LXRα/β-KO mice and measuring plasma cytokine levels. Injection of α-GalCer to WT-mice increased plasma IL-4 and IFN-γ, reaching peak levels at 3 and 24 h, respectively (Fig. 3a), as reported previously^18^. Plasma IL-4 levels at the 3-h time point were lower in LXRβ-KO mice and comparable to control levels in LXRα/β-KO mice (Fig. 3a). Plasma IFN-γ levels at the 24-h time point were also lower in LXRα/β-KO mice and LXRβ-KO mice, and did not increase in LXRα/β-KO mice. Consistent with in vitro studies (Fig. 2a, b), the basal level of plasma IFN-γ at the 0-h time point in LXRα/β-KO mice was higher than that in WT mice. Con-A, a lectin derived from jack beans, also induces IL-4 production by iNKT cells via a mechanism distinct from α-GalCer^21,22^. Intravenous Con-A treatment effectively increased plasma IL-4 levels in WT mice, although to a lesser extent than α-GalCer treatment (Fig. 3b), as reported previously^23^. The induction of IL-4 in LXRα/β-KO mice was much weaker than that in WT mice (Fig. 3b). These findings indicate that cytokine production by iNKT cell stimulation is impaired in LXRα/β-KO mice.

### iNKT cell-mediated antitumor effect is decreased in LXRα/β-KO mice

**Figure 4:**
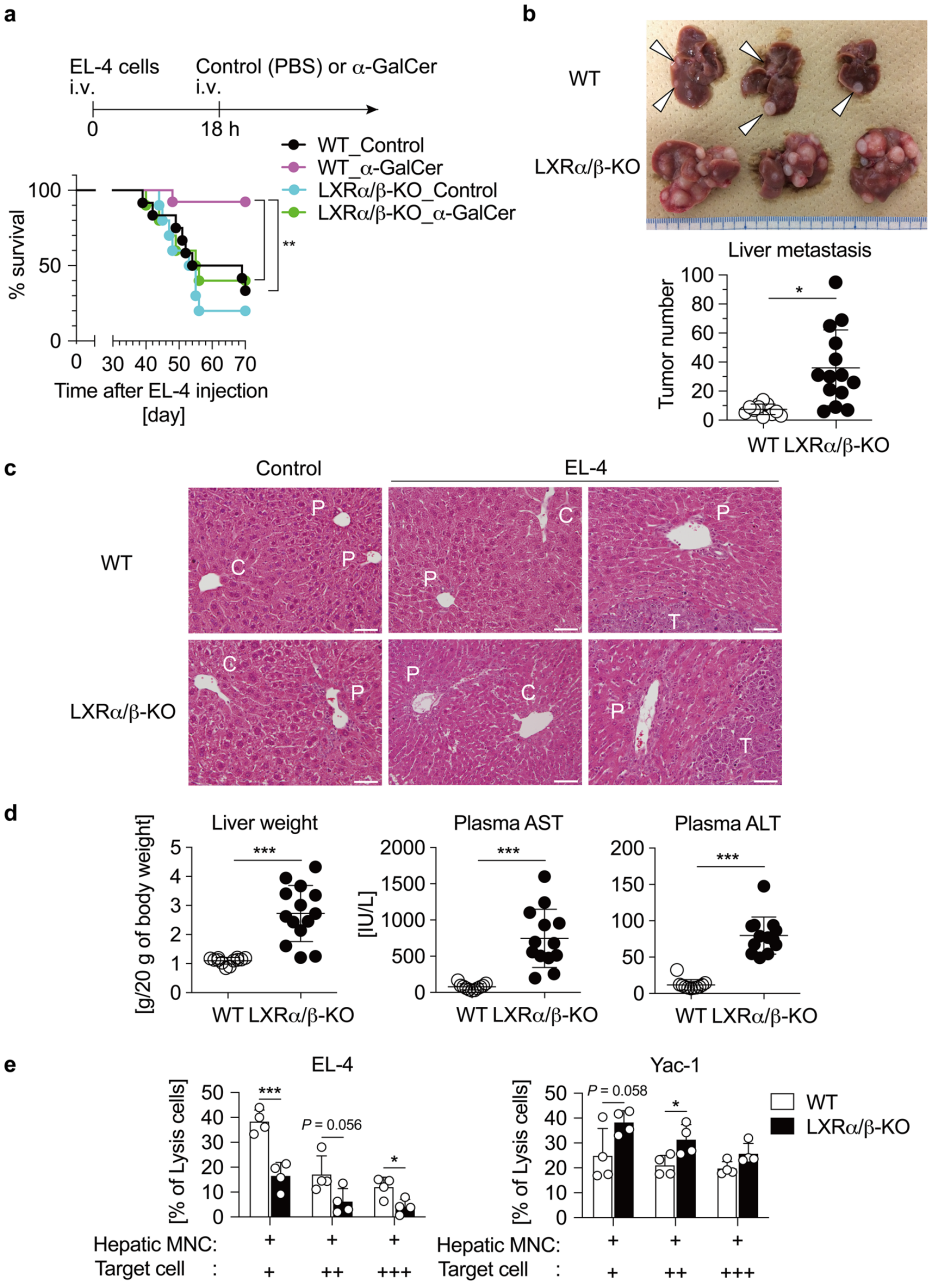
NKT cell-mediated antitumor immunity is impaired in LXRα/β-KO mice. (**a**) Survival rate of mice inoculated with EL-4 tumor cells. WT and LXRα/β-KO mice were injected with EL-4 cells (5 × 10^5^ cells/20 g of body weight), and 18 h later treated with vehicle control (phosphate buffered saline) or α-GalCer (0.1 mg/kg of body weight) intravenously (n = 10–13 for each group). **P < 0.01 (Log-rank test). (**b**) Liver metastasis in mice inoculated with EL-4 cells. The number of metastasis was counted in the liver of WT and LXRα/β-KO mice 28 days after injection of EL-4 cells (1 × 10^6^ cells/20 g of body weight). Arrow heads in the photo indicate metastatic tumors in WT mice. *P < 0.05 (Mann–Whitney’s test). (**c**) Hematoxylin and eosin staining of the liver. The labels C, P and T indicate central vein, portal vein and tumor, respectively. (**d**) Liver weight, plasma aspartate aminotransferase (AST) and alanine aminotransferase (ALT) in mice inoculated with EL-4. ***P < 0.001 (Student’s *t* test). (**e**) Cytotoxicity activity of hepatic MNCs. Hepatic MNCs were isolated form WT and LXRα/β-KO mice, and co-cultured with different concentrations of calcein-labeled EL-4 cells or Yac-1 cells (+ , 5 × 10^4^ cells/well; +  + , 1 × 10^5^ cells/well; +  +  + , 2 × 10^5^ cells/well). After incubation for 4 h, fluorescent intensity in the culture media was measured for cell lysis (n = 4). *P < 0.05, ***P < 0.001 (Student’s *t* test).

NKT cells, particularly in the liver, are involved in antitumor immunity^6^. EL-4 cells are mouse T cell lymphoma-derived cells which express CD1d and when injected into mice are directly killed by a mechanism requiring NKT cells^6^. Survival of WT mice and LXRα/β-KO mice was assessed after EL-4 cell injection. Mice were injected with EL-4 cells intravenously and then treated with α-GalCer for iNKT cell activation. Five of 12 WT mice (33%) without α-GalCer treatment survived at 70 days after EL-4 cell injection (Fig. 4a). iNKT cell stimulation by α-GalCer increased the survival rate of mice to 93% (12 of 13 mice), consistent with our previous report^7^. On the other hand, only 2 of 10 LXRα/β-KO mice (20%) without α-GalCer treatment survived, and α-GalCer treatment did not increase the survival rate significantly (4 of 10 mice (40%); *P* > 0.05). Next, we analyzed metastatic tumors in the liver of α-GalCer-untreated mice 28 days after injection of EL-4 cells. All mice were alive at the 28-day time point (Fig. 4a). We observed several hepatic tumors in both WT and LXRα/β-KO mice (Fig. 4b). Although there was no statistically significant difference in survival between WT and LXRα/β-KO mice (33% vs 20%) in the absence of α-GalCer treatment when monitored for 70 days (Fig. 4a), the tumor number in the liver of LXRα/β-KO mice was much higher than that in WT mice at 28-day time point (Fig. 4b). Liver histology showed more tumor cell infiltrate around the portal vein in the liver of LXRα/β-KO mice compared to WT mice (Fig. 4c). Liver weight, plasma aspartate aminotransferase and alanine aminotransferase levels were increased in EL-4 cell-injected LXRα/β-KO mice (Fig. 4d), indicating that liver metastasis is associated with hepatocyte damage.

Next, we examined NKT cell-mediated antitumor effect in co-culture experiments. We attempted to isolate hepatic iNKT cells from WT and LXRα/β-KO mice but could not obtain enough number of cells, because iNKT cells were severely decreased in LXRα/β-KO mice (Fig. 1d). Then, we isolated hepatic MNCs containing iNKT cells from WT and LXRα/β-KO mice, cultured these cells with calcein-labeled tumor cells, and evaluated tumor cell killing by measuring fluorescence intensity in cultured media. We compared the antitumor effect against NK cell-resistant EL-4 cells and NK cell-sensitive Yac-1 cells^24,25^. The antitumor effect of hepatic MNCs against EL-4 cells was lower in LXRα/β-KO cells when compared to WT cells (Fig. 4e), consistent with the in vivo results (Fig. 4a–c) Antitumor effect against Yac-1 cells was not changed or slightly increased in LXRα/β-KO cells. These findings indicate that NKT cell-mediated antitumor effect is decreased in hepatic MNCs from LXRα/β-KO mice.

### Expression of the MHC class I-like molecule CD1d and the chemokine C-X-C motif ligand (CXCL) 16 is not decreased in the liver of LXRα/β-KO mice

**Figure 5:**
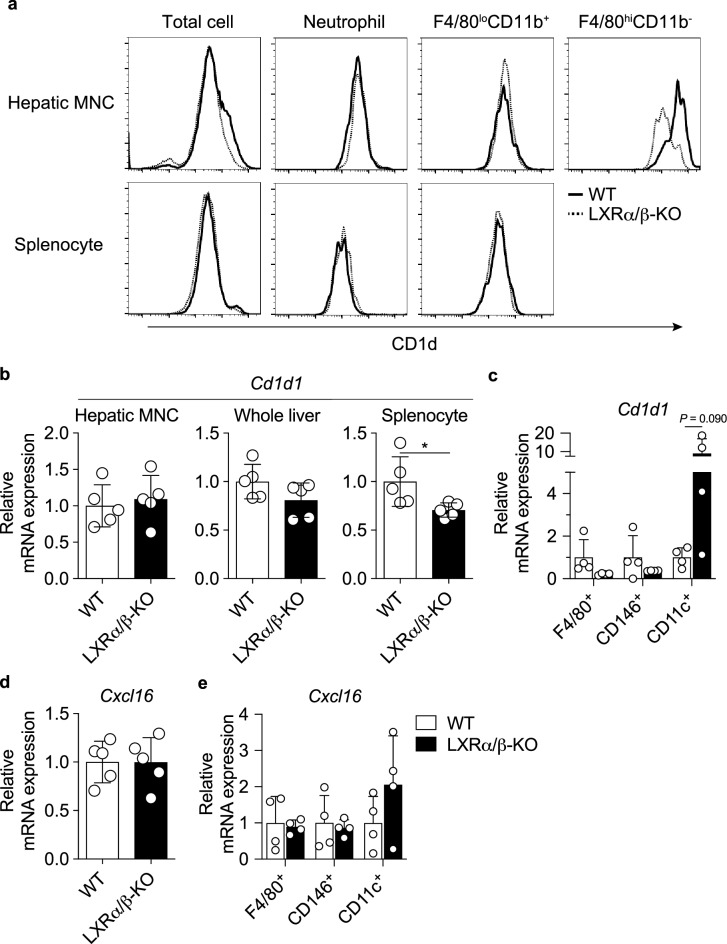
Expression of the MHC class I-like molecule CD1d and the chemokine CXCL16. (**a**) Representative flow cytometry for CD1d expression in hepatic immune cells and splenocytes. Hepatic MNCs and splenocytes were isolated from WT and LXRα/β-KO mice, and stained with APC-conjugated anti-CD1d antibody together with FITC-conjugated anti-F4/80 antibody and Pacific Blue-conjugated anti-CD11b antibody. Expression of CD1d was analyzed in hepatic MNCs, splenocytes, neutrophils (F4/80^−^CD11b^+^), BMDMs (F4/80^lo^CD11b^+^) and resident Kupffer cells (F4/80^hi^CD11b^−^). Similar results were obtained in repeated experiments (n = 4 for each analysis). (**b**) *Cd1d1* mRNA expression in hepatic MNCs, whole liver samples and splenocytes. (**c**) *Cd1d1* mRNA expression in F4/80^+^ Kupffer cells/macrophages, CD146^+^ liver sinusoidal endothelial cells and CD11c^+^ dendritic cells isolated from hepatic MNCs. (**d**) *Cxcl16* mRNA expression in hepatic MNCs. (**e**) *Cxcl16* mRNA expression in F4/80^+^ Kupffer cells/macrophages, CD146^+^ liver sinusoidal endothelial cells and CD11c^+^ dendritic cells isolated from hepatic MNCs. Hepatic MNCs with collagenase digestion, whole liver samples and splenocytes were obtained from WT and LXRα/β-KO mice (n = 5) (**a**, **b** and **d**). Hepatic MNCs were isolated after collagenase perfusion, and F4/80^+^ cells, CD146^+^ cells and CD11c^+^ cells were sorted with magnetic beads. *P < 0.05 (Student’s *t* test).

The MHC class I-like molecule CD1d presents a glycolipid antigen to the invariant TCR of iNKT cells and is essential for iNKT cell development and activation^4^. CD1d expression was unaffected in total hepatic MNCs, neutrophils (F4/80^−^CD11b^+^) or BMDMs (F4/80^lo^CD11b^+^) when comparing WT and LXRα/β-KO mice, while it was slightly decreased in resident Kupffer cells (F4/80^hi^CD11b^−^) from LXRα/β-KO mice (Fig. 5a). Splenic total MNCs, neutrophils and BMDMs had similar CD1d expression in WT and LXRα/β-KO mice. While splenocytes from LXRα/β-KO mice had slightly lower *Cd1d1* expression compared to WT splenocytes, *Cd1d1* mRNA levels did not differ in hepatic MNCs or whole liver between WT and LXRα/β-KO mice (Fig. 5b), suggesting that *Cd1d1* expression is not decreased in a major population of hepatic MNCs and parenchymal cells, 70–80% of which are hepatocytes. We isolated F4/80^+^ Kupffer cells/macrophages, CD146^+^ liver sinusoidal endothelial cells and CD11c^+^ dendritic cells from hepatic MNCs. *Cd1d1* mRNA expression tended to decrease in F4/80^+^ cells and CD146^+^ cells and to increase in CD11c^+^ cells in the liver of LXRα/β-KO mice, although these differences were not statistically significant (Fig. 5c). The decrease of iNKT cells in LXRα/β-KO mice does not appear to be due to deficient CD1d presentation by other immune cells such as dendritic cells. Since CXCL16 in liver sinusoidal epithelial cells and Kupffer cells/macrophages is necessary for the accumulation of iNKT cells, which express the C-X-C motif chemokine receptor 6, and NKT-mediated antitumor immunity in the liver^26–28^. *Cxcl16* mRNA expression was not decreased in hepatic MNCs from LXRα/β-KO mice (Fig. 5d), and also not in F4/80^+^ Kupffer cells/macrophages, CD146^+^ liver sinusoidal endothelial cells or CD11c^+^ dendritic cells from the liver of LXRα/β-KO mice (Fig. 5e). Thus, decreased abundance of iNKT cells in LXRα/β-KO mice are not associated with CXCL16 expression.

### Decreased population of iNKT cells in the thymus of LXRα/β-KO mice

**Figure 6:**
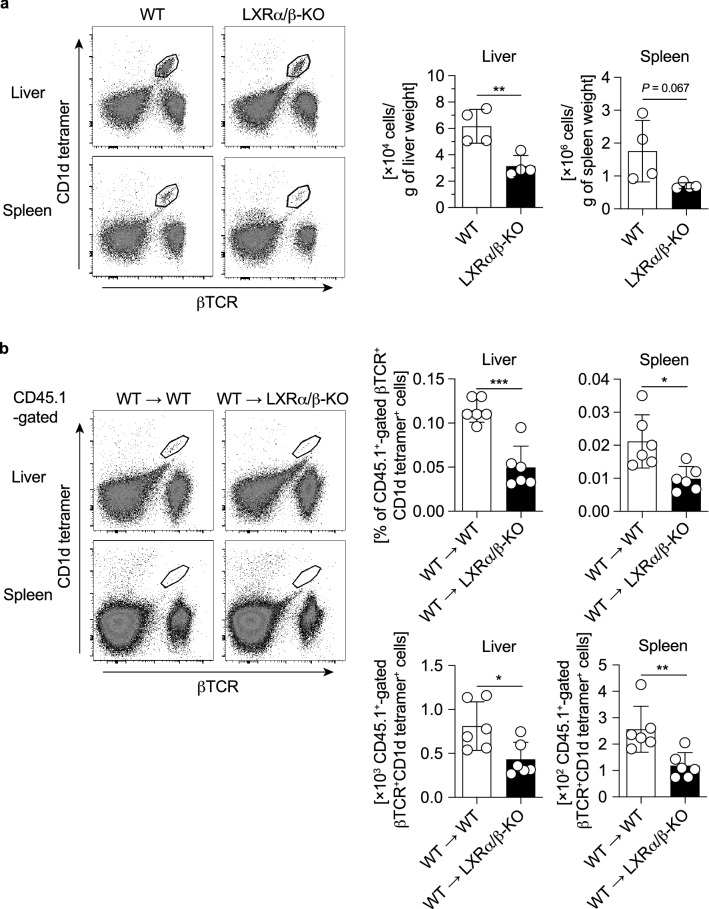
iNKT cells in young mice and bone marrow-transplanted mice. (**a**) The number of iNKT cells in the liver and spleen of young mice. The number of iNKT cells from 4-week-old WT and LXRα/β-KO mice was examined with flow cytometry (n = 4). (**b**) Percentages and numbers of iNKT cells in bone marrow-transplanted mice. Bone marrow cells were isolated from CD45.1 WT mice and transplanted to irradiated CD45.2 WT and CD45.2 LXRα/β-KO mice. After 4 weeks, hepatic MNCs and splenocytes were isolated from recipient WT and LXRα/β-KO mice (n = 6), and analyzed with flow cytometry for iNKT cell populations in CD45.1-gated MNCs. Percentages and cell numbers per tissue weight were shown. *P < 0.05, **P < 0.01, ***P < 0.001 (Student’s *t* test).

We further investigated the effect of LXR deficiency on iNKT cell population in young mice. The number of iNKT cells increases in the liver during development from youth to middle age in mice^29^. We analyzed a population of iNKT cells in the liver and spleen in 4-week-old young mice, and found that iNKT cells were decreased in the liver of LXRα/β-KO mice compared to WT mice (Fig. 6a). A similar tendency was observed in the spleen although it was not statistically significant. Next, we performed bone marrow transplantation experiments utilizing bone marrow cells derived from CD45.1 WT mice to irradiated CD45.2 WT or LXRα/β-KO recipient mice, and found that iNKT cell development from WT bone marrow cells was impaired in both liver and spleen of LXRα/β-KO mice (Fig. 6b). The populations of NK cells and conventional T cells were not affected LXR deficiency (Supplementary Fig. 2). Thus, iNKT cell development may be impaired in the absence of LXRα/β function at a stage following bone marrow exit.

**Figure 7:**
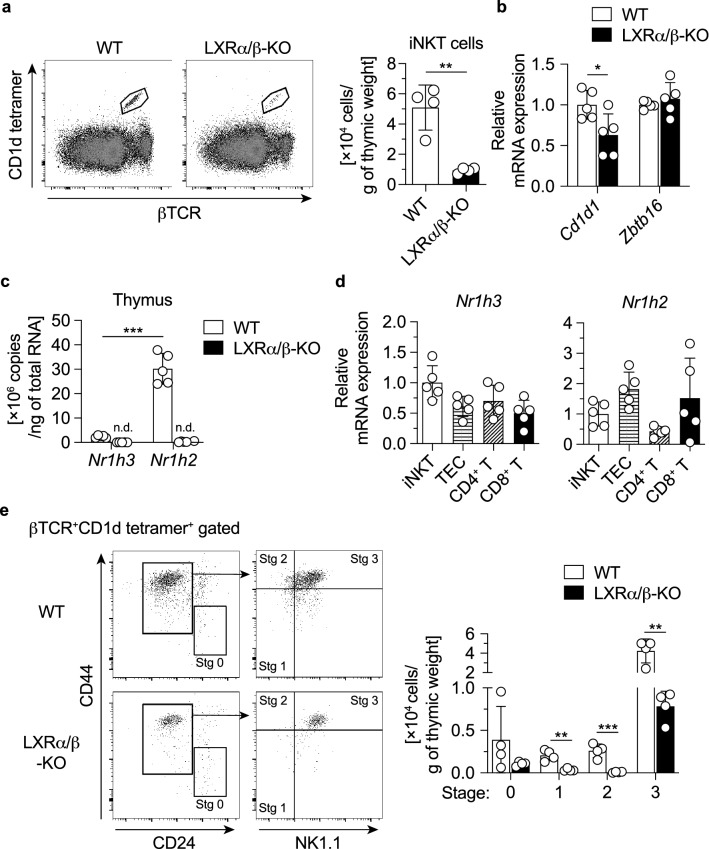
Decreased population of iNKT cells in the thymus of LXRα/β-KO mice. (**a**) Numbers of iNKT cells in thymocytes. (**b**) *Cd1d1* and *Zbtb16* mRNA expression and (**c**) *Nr1h3* and *Nr1h2* mRNA expression in the thymus. (**d**) mRNA expression of *Nr1h3* and *Nr1h2* in iNKT cells, TECs, CD4^+^ T cells and CD8^+^ T cells from the thymus. Thymocytes were stained with PE-conjugated CD1d tetramer, FITC-conjugated anti-βTCR, PE-Cy7-conjugated anti-EpCAM and APC-conjugated anti-CD45 to separate iNKT cells (CD45^+^βTCR^+^CD1d-tetramer^+^ cells) and TECs (EpCAM^+^ cells), or with FITC-conjugated anti-βTCR, PE-conjugated anti-CD4 and PE-Cy7-conjugated anti-CD8a to separate CD4^+^ T cells (CD45^+^βTCR^+^CD4^+^ cells) and CD8^+^ T cells (CD45^+^βTCR^+^CD8a^+^ cells). (**e**) The populations of thymic iNKT cells of stage 0, stage 1, stage 2 and stage 3. Thymocytes were isolated from WT and LXRα/β-KO mice, and stained with FITC-conjugated anti-NK1.1, PE-conjugated CD1d tetramer, PE-Cy5-conjugated anti-βTCR, Brilliant Violet-510-conjugated anti-CD24 and APC-conjugated anti-CD44. iNKT cell stages were determined as stage 0 (CD24^+^CD44^−^), stage 1 (CD24^−^CD44^−^NK1.1^−^), stage 2 (CD24^−^CD44^+^NK1.1^−^) and stage 3 (CD24^−^CD44^+^NK1.1^+^) in the population of βTCR^+^CD1d-tetramer^+^-gated cells (iNKT cells). Stg, stage. *P < 0.05, **P < 0.01, ***P < 0.001 (Student’s *t* test). n.d., not detected (around or below detection limits).

Lymphoid precursors leave the bone marrow and develop into iNKT cells in the thymus^3^. We examined the effect of LXR deficiency on thymic iNKT cell development. The number of iNKT cells and level of *Cd1d1* mRNA expression were decreased in the thymus of LXRα/β-KO mice (Fig. 7a, b). The transcription factor promyelocytic leukemia zinc finger (PLZF) is essential for the development of NKT cell lineage at the early developmental stage^30^. The expression of *Zbtb16* (the gene encoding PLZF) was not altered in LXRα/β-KO mice (Fig. 7b). In thymocytes, *Nr1h2* (the gene encoding LXRβ) was highly expressed compared to *Nr1h3* (the gene encoding LXRα) (Fig. 7c). mRNA levels of *Nr1h3* and *Nr1h2* in sorted thymic iNKT cells were similar to those in thymic epithelial cells (TECs), CD4^+^ T cells and CD8^+^ T cells in the thymus (Fig. 7d). iNKT cells develop through stage 0 (CD24^+^CD44^−^), stage 1 (CD24^−^NK1.1^−^CD44^−^), stage 2 (CD24^−^NK1.1^−^CD44^+^) and stage 3 (CD24^−^NK1.1^+^CD44^+^) in the thymus^3,31,32^. We evaluated the abundance of thymic iNKT cells at these developmental stages in adult mice. The distribution of abundance of iNKT cell stages in WT thymus was similar to that reported previously^32^. Compared to WT mice, stage 1, stage 2 and stage 3 cells were significantly decreased in LXRα/β-KO mice (Fig. 7e). Stage 0 cells tended to be lower in LXRα/β-KO mice, but this difference was not statistically significant. Therefore, iNKT cell development is impaired in the thymus of LXRα/β mice.

## Discussion

In this study, we report that LXRα and LXRβ are essential for iNKT cell-mediated immunity in mice. While MNCs and F4/80^lo^CD11b^+^ cells are increased in the liver of LXRα/β-KO mice^17^, iNKT cells were nearly absent in the liver and spleen of LXRα/β-KO mice (Fig. 1 and Supplementary Fig. 1). We previously reported that the percentage of NKT cells in hepatic MNCs is not different in LXRα-KO mice and WT mice fed a standard diet^18^. In the current study, we calculated cell numbers per liver weight and observed that hepatic NKT cells and iNKT cells are decreased in LXRα-KO mice. LXRβ-KO mice also had a decreased population of iNKT cells in the liver, and LXRα/β-KO mice showed more severe phenotypes (Fig. 1). Consistent with the decreased number of iNKT cells, cytokine production was impaired in LXRα/β-KO hepatic MNCs and splenocytes after stimulation with the NKT cell-specific activator α-GalCer and also in LXRα/β-KO mice treated with α-GalCer or Con-A (Figs. 2 and 3). The percentage of iNKT cells in hepatic MNCs is decreased in LXRα-KO mice fed high-fat and high-cholesterol diet and the response to α-GalCer is impaired in these mice^18^. Con-A-induced hepatitis is attenuated in LXRα-KO mice and is exacerbated in constitutively active LXRα knock-in mice^33^. These findings indicate that LXRα and also LXRβ are involved in hepatic iNKT cell-mediated immune responses.

Cytokine production (such as IL-4, IFN-γ and TNF-α) induced by the iNKT cell activator α-GalCer was impaired in LXRα/β-KO hepatic MNCs and in LXRα/β-KO mice (Figs. 2 and 3), supporting the importance of LXR in iNKT immunity. Interestingly, hepatic *Ifng* mRNA expression, IFN-γ protein levels in conditioned media of hepatic MNCs and plasma IFN-γ levels were higher in LXRα/β-KO mice than WT mice in the absence of α-GalCer stimulation (Figs. 2 and 3). It has been reported that IFN-γ^+^CD4^+^ T cells and IFN-γ^+^CD8^+^ T cells are increased in LXRβ-KO mice^34^ and that LXR ligand activation decreases IFN-γ expression in CD4^+^ T cells^35^, suggesting that CD4^+^ and/or CD8^+^ T cells are involved in the increased expression of IFN-γ in the liver of LXRα/β-KO mice. On the other hand, *Ifng* mRNA expression in splenocytes was not affected by LXR deficiency (Fig. 2). Treatment with α-GalCer did not change *Ifng* expression at 6 h in both WT and LXRα/β-KO splenocytes. Basal and α-GalCer-induced *Ifng* expression may be influenced by tissue-selective factors, such as metabolic environment and distinct immune cell composition.

Importantly, LXRα/β-KO mice were incompetent in mounting antitumor immunity against EL-4 tumor cells in in vivo and in vitro experiments (Fig. 4). LXR antitumor effect has also been reported in mice and humans via a mechanism in which induction of the LXR target apolipoprotein E depletes myeloid-derived suppressor cells and activates cytotoxic T cells^36^. In contrast, LXRα activation inhibits dendritic cell-dependent antitumor responses^37^, although LXRα and LXRβ are necessary for dendritic cell chemotaxis^38,39^. Thus, LXRs function in antitumor immunity in an immune cell type-dependent manner. In contrast to the effect against NK cell-resistant EL-4 cells, antitumor effect against NK cell-sensitive Yac-1 cells was not repressed in LXRα/β-KO cells (Fig. 4). We examined the LXR antitumor effect in mice and hepatic MNCs, which contain iNKT cells and other immune cells. Depletion of Kupffer cells decreases liver metastases in EL-4-injected mice^40^. iNKT cells are essential for antitumor immunity against EL-4 cells^41^. These findings suggest that repressed antitumor immunity in the liver of LXRα/β-KO mice is due to impaired iNKT cell-mediated immunity, including a decreased population of these cells. However, we could not compare immune functions of hepatic iNKT cells isolated from WT and LXRα/β-KO mice because iNKT cells were nearly absent in the liver of LXRα/β-KO mice (Fig. 1). In addition, the involvement of other immune cells and parenchymal cells in LXR-mediated antitumor immunity cannot be ruled out. Further studies, such as analysis of mice with iNKT cell-specific deletion of LXR and evaluation of iNKT cells derived from pluripotent stem cells with LXR deletion, will help elucidate the role of LXR in hepatic immunity directly or indirectly regulated by iNKT cells. Recently, several clinical trials utilizing the antitumor activity of iNKT cells have been reported for the treatment of cancer, including as melanoma^42^, non-small cell lung cancer^43,44^, head and neck cancer^45^, and neuroblastoma^11^. Thus, LXR may be used as a therapeutic target in iNKT cell antitumor immunotherapy.

iNKT cells were decreased in the liver and spleen of both young and adult LXRα/β-KO mice (Figs. 1, 6, 7 and Supplementary Fig. 1) and also in those tissues of LXRα/β-KO mice transplanted with WT bone marrow (Fig. 6). Analysis of developmental stages of thymic iNKT cells showed a decrease in stage 1, stage 2 and stage 3 iNKT cells in LXRα/β-KO mice (Fig. 7). In addition, stage 3 iNKT cells were decreased in the liver and spleen of LXRα/β-KO mice (Fig. 1 and Supplementary Fig. 1). These finding suggest that LXR deficiency disturbs iNKT cell development or distribution in both thymus and peripheral organs and leads to a marked decrease in iNKT cells in the liver. PLZF is necessary for development of the NKT cell lineage^30^, and its expression is induced by the transcription factor early growth response 2 at the developmental stage 0^46^. The expression of *Zbtb16* (the gene coding PLZF) was not altered in the thymus of LXRα/β-KO mice (Fig. 7). These findings indicate that LXRs are involved in NKT cell development at the pathway downstream of or independent of PLZF signaling. A recent report by Chan et al*.* shows that LXRα and LXRβ are involved in thymic T cell development through mechanisms of action on multiple cell types, including TECs and thymocytes^47^. Developmental stages of iNKT cells are partly overlapped with those of conventional T cells but are distinct such as in NK1.1 expression^3^. LXR-dependent mechanisms for conventional T cell development may also be involved in iNKT cell development in the thymus. iNKT cells were decreased in the liver and spleen of LXRα/β-KO mice transplanted with WT bone marrow compared to WT mice transplanted with WT bone marrow (Fig. 6), but the number of conventional T cells in these tissues was not different (Supplementary Fig. 2). These findings suggest that LXR in non-bone marrow-derived cells plays a role in development of iNKT cells. Generation of CD45.1 LXRα/β-KO mice and bone marrow transplantation experiments utilizing these mice will be helpful for evaluating the intrinsic role of LXR in bone marrow-derived cells. iNKT cell development during animal development should also be further investigated in mice with cell-specific LXR deletion.

The population of hepatic iNKT cells in adult LXRα/β-KO mice was markedly decreased compared to young LXRα/β-KO mice (Figs. 1 and 6). LXRα/β-KO mice have increased F4/80^lo^CD11b^+^ Kupffer cells/macrophages in the liver and also increased cholesterol content in hepatic MNCs^17^. High-fat and high-cholesterol diet feeding increased hepatic MNCs and F4/80^+^CD68^+^CD11b^+^ Kupffer cells/macrophages in the liver of LXRα-KO mice^18^. Interestingly, these mice demonstrate decreased iNKT cell numbers and cytokine production. Activation of Kupffer cells/macrophages decreases the number of iNKT cells by inducing cell death in mouse models of hepatic steatosis and nonalcoholic steatohepatitis^48,49^. Deletion of LXRα/β in macrophages induces lipid accumulation in the thymus but does not interfere with T cell production^47^. NKT cells develop in the thymus and also in other organs, such as the liver^20^. Increased and activated Kupffer cells/macrophages might be involved in iNKT suppression in the liver of LXRα/β-KO mice. The immune cell communication between Kupffer cells/macrophages and iNKT cells should be further investigated.

In conclusion, we have found that LXRs play an essential role in iNKT cell-mediated immunity, including hepatic antitumor activity. Our findings provide a mechanistic insight into LXR-targeted immunotherapy.

## Materials and methods

### Compounds

α-GalCer was purchased from Funakoshi Co. (Tokyo, Japan) and Con-A was from Vector Laboratories (Burlingame, CA).

### Mice and treatment

WT C57BL/6 J mice were purchased from Nihon CLEA (Tokyo, Japan), and CD45.1 (WT) B6-Ly5.1 mice were from Sankyo Labo Service Corporation, Inc., Tokyo, Japan. *Nr1h3*^-/-^ (*Lxrα*^-/-^, LXRα-KO), *Nr1h2*^-/-^ (*Lxrβ*^-/-^, LXRβ-KO) and *Nh1h3*^-/-^; *Nr1h2*^-/-^ (*Lxrα*^-/-^; *Lxrβ*^-/-^, LXRα/β-KO) mice were kindly provided by Dr. David J. Mangelsdorf (University of Texas Southwestern Medical Center at Dallas, TX)^50,51^, and were backcrossed with C57BL/6 J mice for at least ten generations. Mice were maintained under controlled temperature (23 ± 1 °C) and humidity (45–55%) with a 12-h light, 12-h dark cycles, and with free access to water and chow (CE-2; Nihon CLEA). Young male mice (4 weeks old) and adult male mice (12–16 weeks old) were used for experiments. To activate NKT cells in vivo, mice were injected intravenously with α-GalCer (0.1 mg/kg of body weight) or Con-A (12.5 mg/kg of body weight) as reported previously^18^. Tissue samples were collected after euthanization with CO_2_ inhalation. All animal experiments were performed according to the protocols, which adhered to the Nihon University Rules concerning Animal Care and Use, approved by Nihon University Animal Care and Use Committee (AP14M066, AP15M027, AP17M014, AP17M015, AP17M073, AP18MED007-1), and conformed to the ARRIVE guidelines.

### Isolation of hepatic MNCs, splenocytes and thymocytes

MNCs were isolated with or without collagenase digestion and separated from parenchymal cells with Percoll solution (Sigma-Aldrich, St. Louis, MO) as reported previously^17,18^. For detection of lymphocytes including iNKT cells, MNCs were isolated without collagenase digestion. For detection of Kupffer cells and myeloid cells, MNCs were isolated with the 0.5 mg/mL collagenase digestion (Wako Pure Chemical Industries, Osaka, Japan) for 25 min at 37 °C. F4/80^+^ cells, CD146^+^ cells and CD11c^+^ cells were isolated from the liver with collagenase perfusion using QuadroMACS sorting system (Miltenyi Biotec, Bergisch Gladbach, Germany) with Anti-F4/80 MicroBeads, CD146 (LSEC) MicroBeads and CD11c MicroBeads UltraPure (Miltenyi Biotec).

Splenocytes or thymocytes were isolated after filtration with 40 μm nylon mesh and removal of red blood cells. For NKT cell activation in vitro, cells were isolated with collagenase digestion and treated α-GalCer for 6 h for gene expression analysis, or 16 h for enzyme-linked immunosorbent assay.

### Flow cytometric analysis

For detection of NK cells (βTCR^−^NK1.1^+^) or NKT cells (βTCR^+^NK1.1^+^), cells were isolated without collagenase digestion, incubated with LIVE/DEAD Fixable Near-IR Dead Cell Stain Kit (Thermo Fisher Scientific, Waltham, MA) and anti-CD16/32 Fc blocker (Thermo Fisher Scientific, cat No.14-0161, clone No. 93, RRID: AB_467133, 30-fold dilution), and stained with fluorescein isothiocyanate (FITC)-conjugated anti-βTCR antibody (Thermo Fisher Scientific, cat No.11-5962, clone No. H57-597, RRID: AB_465323, tenfold dilution), phycoerythrin (PE)-conjugated anti-NK1.1 antibody (Thermo Fisher Scientific, cat No.12-5941, clone No. PK136, RRID: AB_466050, 30-fold dilution), biotin-conjugated anti-CD69 antibody (Thermo Fisher Scientific, cat No.13-0691, clone No. H1.2F3, RRID: AB_466495, 30-fold dilution) and PE-Cy5-streptavidin (Thermo Fisher Scientific).

For detection of iNKT cells (βTCR^+^CD1d-tetramer^+^), cells were isolated without collagenase digestion, incubated with LIVE/DEAD Fixable Near-IR Dead Cell Stain Kit and anti-CD16/32 Fc blocker, and stained with FITC-conjugated anti-NK1.1 antibody (Thermo Fisher Scientific, cat No.11-5941, clone No. PK136, RRID: AB_465318, tenfold dilution), PE-conjugated CD1d tetramer complexed with α-GalCer (Medical & Biological Laboratories Co., Nagoya, Japan, cat No. TS-MCD-1, 20-fold dilution), PE-Cy5-conjugated βTCR antibody (Thermo Fisher Scientific, cat No.15-5961, clone No. H57-597, RRID: AB_468816, 30-fold dilution), allophycocyanin-conjugated (APC-conjugated) anti-CD44 antibody (BioLegend, San Diego, CA, cat No.103012, clone No. IM7, RRID: AB_312963, 50-fold dilution) and/or Brilliant Violet 510-conjugated CD24 antibody (BioLegend, cat No.101831, clone No. M1/69, RRID: AB_2563894, 50-fold dilution).

For detection of CD1d-expressing antigen-presenting cells in hepatic MNCs such as neutrophils (F4/80^−^CD11b^+^), BMDMs (F4/80^lo^CD11b^+^) and resident Kupffer cells (F4/80^hi^CD11b^−^), cells were isolated with collagenase digestion, incubated with LIVE/DEAD Fixable Near-IR Dead Cell Stain Kit and anti-CD16/32 Fc blocker, and stained with FITC-conjugated F4/80 antibody (Thermo Fisher Scientific, cat No.11-4801, clone No. BM8, RRID: AB_2637191, tenfold dilution), Pacific Blue-conjugated anti-CD11b antibody (BioLegend, cat No.101224, clone No. M1/70, RRID: AB_755986, 50-fold dilution) and APC-conjugated anti-CD1d antibody (BioLegend, cat No.123522, clone No. 1B1, RRID: AB_2715920, 50-fold dilution).

For bone marrow transplantation analysis, cells were isolated without collagenase digestion, incubated with LIVE/DEAD Fixable Near-IR Dead Cell Stain Kit and anti-CD16/32 Fc blocker, and stained with FITC-conjugated anti-NK1.1 antibody, PE-conjugated CD1d tetramer complexed with α-GalCer, PE-Cy5-conjugated βTCR antibody, APC-conjugated CD45.1 antibody (BioLegend, cat No.110714, clone No. A20, RRID: AB_313503, 30-fold dilution) and Pacific Blue-conjugated anti-CD45.2 antibody (BioLegend, cat No.109820, clone No. 104, RRID: AB_492872, 30-fold dilution) to identify WT-derived iNKT cells (CD45.1^+^CD45.2^−^βTCR^+^CD1d tetramer^+^ cells).

The stained cells were analyzed with BD FACSVerse flow cytometer (BD Biosciences, San Jose, CA) and cell proportions were with FlowJo 10.4 software (Tree Star, Ashland, OR).

For the sorting of iNKT cells and TECs from WT mice, thymocytes were incubated with LIVE/DEAD Fixable Near-IR Dead Cell Stain Kit and anti-CD16/32 Fc blocker, and stained with FITC-conjugated anti-βTCR antibody, PE-conjugated CD1d tetramer complexed with α-GalCer, and PE-Cy7-conjugated CD326 (EpCAM) antibody (BioLegend, cat No.118216, clone No. G8.8, RRID: AB_1236471, 50-fold dilution) and APC-conjugated CD45 antibody (BioLegend, cat No.103112, clone No. 30-F11, RRID: AB_312977, 50-fold dilution) to identify iNKT cells (CD45^+^ βTCR^+^CD1d tetramer^+^ cells) or TECs (EpCAM^+^ cells). For the sorting of CD4^+^ T cells and CD8^+^ T cells, cells were incubated with LIVE/DEAD Fixable Near-IR Dead Cell Stain Kit and anti-CD16/32 Fc blocker, and stained with FITC-conjugated anti-βTCR antibody, PE-conjugated CD4 antibody (BioLegend, cat No.100408, clone No. GK1.5, RRID: AB_312693, 50-fold dilution), PE-Cy7-conjugated CD8a antibody (BioLegend, cat No.100722, clone No. 53-6.7, RRID: AB_312761, 50-fold dilution) and APC-conjugated CD45 antibody (BioLegend, cat No.103112, clone No. 30-F11, RRID: AB_312977, 50-fold dilution) to identify CD4^+^ T cells (CD45^+^βTCR^+^CD4^+^ cells) and CD8^+^ T cells (CD45^+^βTCR^+^CD8a^+^ cells). Cells (100,000 cells for each cell types) were sorted using cell sorter (FACSAria, BD Biosciences), confirmed with the > 90% purity for each cell types and utilized for gene expression analysis.

### Reverse transcription and quantitative real-time polymerase chain reaction

Total RNA was isolated from hepatic MNCs, splenocytes or thymocytes with RNAiso Plus reagent (TaKaRa Bio Inc., Otsu, Japan), and cDNA was synthesized with ImProm-II reverse transcription system (Promega Corporation, Madison, WI). Quantitative real-time polymerase chain reaction was performed with the StepOnePlus Real-time PCR system (Thermo Fisher Scientific) using Power SYBR Green PCR Master Mix reagent (Thermo Fisher Scientific). The mouse primer sequence was as follows: *Cd1d1* (the gene encoding CD1d), 5′-AGG TCT GGG GAC AAT CTG AA-3′ and 5′-ATG GCT TCG TGA AGC TGA TG-3′; *Cxcl16* (the gene encoding CXCL16), 5′-ACC AGT GGG TCC GTG AAC TA-3′ and 5′-CCT CAG GGG TCT GGG TAC TG-3′; *Zbtb16* (the gene encoding PLZF), 5′-ACC ACA CGG GCT TGT GTA AA-3′ and 5′-ACG GTA CCT GGC AGC AAT AC-3′. The primers of *Il4* (the gene encoding IL-4), *Ifng* (the gene encoding IFN-γ), *Tnf* (the gene encoding TNF-α), *Nr1h3* (the gene encoding LXRα), *Nr1h2* (the gene encoding LXRβ), *Abca1* (the gene encoding ABCA1) and *Ppib* (the gene encoding Cyclophilin B) were reported previously^18,52–54^. The calculated RNA values were normalized with *Ppib* mRNA levels.

### Plasma analysis

Plasma aspartate aminotransferase and alanine aminotransferase levels were measured with GOT·GPT CII-Test Wako (Wako Pure Chemical Industries). Plasma IL-4 and IFN-γ levels were measured with enzyme-linked immunosorbent assay kits (R&D Systems, Minneapolis, MN).

### Hepatic metastasis of EL-4 cells

EL-4 mouse T lymphoma cells (5 × 10^5^ cells/20 g of body weight for the survival analysis or 1 × 10^6^ cells/20 g of body weight for other analyses) were suspended in phosphate buffered saline and intravenously injected to mice. After 18 h, mice were injected intravenously with phosphate buffered saline or α-GalCer (0.1 mg/kg of body weight) and analyzed for survival rate. For other analyses, 28 days after EL-4 administration, hepatic tumor metastasis was counted, liver tissues were stained with hematoxylin and eosin, and plasma aspartate aminotransferase and alanine aminotransferase levels were measured with GOT·GPT CII-Test Wako.

### Cytotoxicity assay

Hepatic MNCs (5 × 10^6^ cells per each well) were isolated by collagenase digestion and plated in a 96-well black plate. Target tumor cells (NK cell-resistant EL-4 or NK cell-sensitive Yac-1 cells)^24,25,55^ were labeled with 5 µg/mL calcein-AM (Sigma-Aldrich) and co-cultured with hepatic MNCs at the different concentration (5 × 10^4^, 1 × 10^5^, or 2 × 10^5^ cells/well). After incubation for 4 h, fluorescent intensity (excitation wavelength: 495 nm, emission wavelength: 515 nm) of culture media was measured with a FlexStation 3 microplate reader (Molecular Devices, Sunnyvale, CA).

### Bone marrow transplantation

Mice were lethally irradiated (10 Gy) using MBR-1512R-3 (Hitachi Medical Corporation, Tokyo, Japan), and injected intravenously with bone marrow cells (1 × 10^7^ cells per 20 g of body weight) isolated from donor B6-Ly5.1 mice. After 4 weeks, hepatic MNCs and splenocytes were isolated from recipient mice, and iNKT cell frequency was analyzed using FACSVerse flow cytometer.

### Statistical analysis

All data are indicated as means ± S.D. We performed one-way analysis of variance (ANOVA) followed by Tukey’s multiple comparisons to analyze data of more than two groups, unpaired Student’s *t* test or Mann–Whitney’s test to compare two groups, two-way ANOVA to analyze the influence of two different factors, and Log-rank test for Kaplan–Meier’s survival curves using Prism 9 (GraphPad Software, La Jolla, CA).

## Supplementary Information

Supplementary Figures.

## References

[1] Seki S, Nakashima H, Nakashima M, Kinoshita M (2011). Antitumor immunity produced by the liver Kupffer cells, NK cells, NKT cells, and CD8^+^CD122^+^T cells. Clin. Dev. Immunol..

[2] Heymann F, Tacke F (2016). Immunology in the liver—From homeostasis to disease. Nat. Rev. Gastroenterol. Hepatol..

[3] Bennstein SB (2018). Unraveling natural killer T-cells development. Front. Immunol..

[4] Bendelac A, Savage PB, Teyton L (2007). The biology of NKT cells. Annu. Rev. Immunol..

[5] Takeda K (2000). Critical contribution of liver natural killer T cells to a murine model of hepatitis. Proc. Natl. Acad. Sci. U. S. A..

[6] Crowe NY (2005). Differential antitumor immunity mediated by NKT cell subsets in vivo. J. Exp. Med..

[7] Inui T (2005). Neutralization of tumor necrosis factor abrogates hepatic failure induced by alpha-galactosylceramide without attenuating its antitumor effect in aged mice. J. Hepatol..

[8] Nakashima H (2006). Activation of mouse natural killer T cells accelerates liver regeneration after partial hepatectomy. Gastroenterology.

[9] Mossanen JC (2019). CXCR6 Inhibits hepatocarcinogenesis by promoting natural killer T- and CD4^+^ T-cell-dependent control of senescence. Gastroenterology.

[10] Kriegsmann K, Kriegsmann M, von Bergwelt-Baildon M, Cremer M, Witzens-Harig M (2018). NKT cells—New players in CAR cell immunotherapy?. Eur. J. Haematol..

[11] Heczey A (2020). Anti-GD2 CAR-NKT cells in patients with relapsed or refractory neuroblastoma: An interim analysis. Nat. Med..

[12] Tontonoz P, Mangelsdorf DJ (2003). Liver X receptor signaling pathways in cardiovascular disease. Mol. Endocrinol..

[13] Joseph SB, Castrillo A, Laffitte BA, Mangelsdorf DJ, Tontonoz P (2003). Reciprocal regulation of inflammation and lipid metabolism by liver X receptors. Nat. Med..

[14] Ghisletti S (2007). Parallel SUMOylation-dependent pathways mediate gene- and signal-specific transrepression by LXRs and PPAR gamma. Mol. Cell.

[15] Ito A (2015). LXRs link metabolism to inflammation through Abca1-dependent regulation of membrane composition and TLR signaling. Elife.

[16] Thomas DG (2018). LXR suppresses inflammatory gene expression and neutrophil migration through cis-repression and cholesterol efflux. Cell Rep..

[17] Endo-Umeda K (2018). Liver X receptors regulate hepatic F4/80^+^CD11b^+^ Kupffer cells/macrophages and innate immune responses in mice. Sci. Rep..

[18] Endo-Umeda K, Nakashima H, Umeda N, Seki S, Makishima M (2018). Dysregulation of Kupffer cells/macrophages and natural killer T cells in steatohepatitis in LXRalpha knockout male mice. Endocrinology.

[19] Ziegler SF, Ramsdell F, Alderson MR (1994). The activation antigen CD69. Stem Cells.

[20] Dashtsoodol N (2017). Alternative pathway for the development of Valpha14^+^ NKT cells directly from CD4^-^CD8^-^ thymocytes that bypasses the CD4^+^CD8^+^ stage. Nat. Immunol..

[21] Kaneko Y (2000). Augmentation of Valpha14 NKT cell-mediated cytotoxicity by interleukin 4 in an autocrine mechanism resulting in the development of concanavalin A-induced hepatitis. J. Exp. Med..

[22] Nakashima H (2008). Superoxide produced by Kupffer cells is an essential effector in concanavalin A-induced hepatitis in mice. Hepatology.

[23] Umeda N (2019). Frontline science: Concanavalin A-induced acute hepatitis is attenuated in vitamin D receptor knockout mice with decreased immune cell function. J. Leukoc. Biol..

[24] Seki S (1997). Antimetastatic effect of NK1^+^ T cells on experimental haematogenous tumour metastases in the liver and lungs of mice. Immunology.

[25] Kawarabayashi N (2010). Immunosuppression in the livers of mice with obstructive jaundice participates in their susceptibility to bacterial infection and tumor metastasis. Shock.

[26] Wehr A (2013). Chemokine receptor CXCR6-dependent hepatic NK T Cell accumulation promotes inflammation and liver fibrosis. J. Immunol..

[27] Geissmann F (2005). Intravascular immune surveillance by CXCR6^+^ NKT cells patrolling liver sinusoids. PLoS Biol..

[28] Ma C (2018). Gut microbiome-mediated bile acid metabolism regulates liver cancer via NKT cells. Science.

[29] Tsukahara A (1997). Mouse liver T cells: Their change with aging and in comparison with peripheral T cells. Hepatology.

[30] Savage AK (2008). The transcription factor PLZF directs the effector program of the NKT cell lineage. Immunity.

[31] Benlagha K, Kyin T, Beavis A, Teyton L, Bendelac A (2002). A thymic precursor to the NK T cell lineage. Science.

[32] Shimizu K (2019). Eomes transcription factor is required for the development and differentiation of invariant NKT cells. Commun. Biol..

[33] Gao L (2020). Activation of liver X receptor alpha sensitizes mice to T-cell mediated hepatitis. Hepatol. Commun..

[34] Bensinger SJ (2008). LXR signaling couples sterol metabolism to proliferation in the acquired immune response. Cell.

[35] Solt LA, Kamenecka TM, Burris TP (2012). LXR-mediated inhibition of CD4^+^ T helper cells. PLoS ONE.

[36] Tavazoie MF (2018). LXR/ApoE activation restricts innate immune suppression in cancer. Cell.

[37] Villablanca EJ (2010). Tumor-mediated liver X receptor-alpha activation inhibits CC chemokine receptor-7 expression on dendritic cells and dampens antitumor responses. Nat. Med..

[38] Feig JE (2010). LXR promotes the maximal egress of monocyte-derived cells from mouse aortic plaques during atherosclerosis regression. J. Clin. Invest..

[39] Beceiro S (2018). Liver X receptor nuclear receptors are transcriptional regulators of dendritic cell chemotaxis. Mol. Cell. Biol..

[40] Ikarashi M (2013). Distinct development and functions of resident and recruited liver Kupffer cells/macrophages. J. Leukoc. Biol..

[41] Bassiri H (2014). iNKT cell cytotoxic responses control T-lymphoma growth in vitro and in vivo. Cancer Immunol. Res..

[42] Exley MA (2017). Adoptive transfer of invariant NKT cells as immunotherapy for advanced melanoma: A phase I clinical trial. Clin. Cancer Res..

[43] Motohashi S (2006). A phase I study of in vitro expanded natural killer T cells in patients with advanced and recurrent non-small cell lung cancer. Clin. Cancer Res..

[44] Motohashi S (2009). A phase I-II study of alpha-galactosylceramide-pulsed IL-2/GM-CSF-cultured peripheral blood mononuclear cells in patients with advanced and recurrent non-small cell lung cancer. J. Immunol..

[45] Uchida T (2008). Phase I study of alpha-galactosylceramide-pulsed antigen presenting cells administration to the nasal submucosa in unresectable or recurrent head and neck cancer. Cancer Immunol. Immunother..

[46] Seiler MP (2012). Elevated and sustained expression of the transcription factors Egr1 and Egr2 controls NKT lineage differentiation in response to TCR signaling. Nat. Immunol..

[47] Chan CT (2020). Liver X receptors are required for thymic resilience and T cell output. J. Exp. Med..

[48] Kremer M (2010). Kupffer cell and interleukin-12-dependent loss of natural killer T cells in hepatosteatosis. Hepatology.

[49] Tang T, Sui Y, Lian M, Li Z, Hua J (2013). Pro-inflammatory activated Kupffer cells by lipids induce hepatic NKT cells deficiency through activation-induced cell death. PLoS ONE.

[50] Peet DJ (1998). Cholesterol and bile acid metabolism are impaired in mice lacking the nuclear oxysterol receptor LXR alpha. Cell.

[51] Repa JJ (2000). Regulation of absorption and ABC1-mediated efflux of cholesterol by RXR heterodimers. Science.

[52] Cummins CL (2006). Liver X receptors regulate adrenal cholesterol balance. J. Clin. Invest..

[53] Ogura M (2009). Vitamin D_3_ modulates the expression of bile acid regulatory genes and represses inflammation in bile duct-ligated mice. J. Pharmacol. Exp. Ther..

[54] Nunomura S, Endo K, Makishima M, Ra C (2010). Oxysterol represses high-affinity IgE receptor-stimulated mast cell activation in Liver X receptor-dependent and -independent manners. FEBS Lett..

[55] Hashimoto W (1995). Cytotoxic NK1.1 Ag+ alpha beta T cells with intermediate TCR induced in the liver of mice by IL-12. J. Immunol..

