# Supplementary material for: Liver X receptors regulate natural killer T cell population and antitumor activity in the liver of mice
Source: Sci Rep. 2021 Nov 19;11:22595. doi: 10.1038/s41598-021-02062-z (PMC8604965; doi:10.1038/s41598-021-02062-z)
Supplement: Supplementary file 1 — Supplementary Figures. [file 41598_2021_2062_MOESM1_ESM.pdf]

Supplementary information

Liver X receptors regulate natural killer T cell population and antitumor activity in the liver of mice

Kaori Endo-Umeda,[1] Hiroyuki Nakashima,[2] Shigeyuki Uno,[1] Shota Toyoshima,[3,5] Naoki Umeda,[1] Shihoko Komine-Aizawa,[4] Shuhji Seki,[2] and Makoto Makishima[1]

[1]Division of Biochemistry, Department of Biomedical Sciences, Nihon University School of Medicine, Itabashi-ku, Tokyo, Japan

[2]Department of Immunology and Microbiology, National Defense Medical College, Tokorozawa, Saitama, Japan

[3]Allergy and Immunology Research Project Team, Research Institute of Medical Science, Center for Institutional Research and Medical Education, Nihon University School of Medicine, Itabashi-ku, Tokyo, Japan

[4]Division of Microbiology, Department of Pathology and Microbiology, Nihon University School of Medicine, Itabashi-ku, Tokyo, Japan

[5]Department of Biochemistry and Molecular Biology, Nippon Medical School, Bunkyo-ku, Tokyo, Japan

Correspondence to: Makoto Makishima (email: makishima.makoto@nihon-u.ac.jp)

Figure legends

Supplementary Figure 1. Decreased population of iNKT cells in the spleen of LXRα/β-KO mice. (**a**) Representative flow cytometry for NKT cells (NK1.1^{+}βTCR^{+}) and NK cells (NK1.1^{+}βTCR^{-}) in splenic MNCs. (**b**) Numbers of NKT cells and NK cells. (**c**) Representative flow cytometry for iNKT cells (βTCR^{+}CD1d-tetramer^{+}). (d) Numbers of iNKT cells. (**e**) Representative flow cytometry for CD69 expression in NK1.1^{+}βTCR^{+}-gated NKT cells and NK1.1^{+}βTCR^{-}-gated NK cells. (**f**) Percentages of CD69-positive cells in NKT cells and NK cells. (**g**) Percentage of NK1.1^{-}CD44^{-} cells (stage 1), NK1.1^{-}CD44^{+} cells (stage 2) and NK1.1^{+}CD44^{+} cells (stage 3) in iNKT cells. Splenocytes were isolated from WT, LXRα-KO, LXRβ-KO and LXRα/β-KO mice and stained with FITC-conjugated anti-βTCR and PE-conjugated anti-NK1.1 (**a**, **b**, **d** and **e**), PE-conjugated CD1d tetramer and PE-Cy5-conjugated anti-βTCR (**c** and **d**) or with FITC-conjugated anti-NK1.1, PE-conjugated CD1d tetramer, PE-Cy5-conjugated anti-βTCR and APC-conjugated anti-CD44 (**g**) (n = 4). **(a-f)** *$P < 0.05$, **$P < 0.01$, ***$P < 0.001$ (one-way ANOVA followed by Tukey's multiple comparisons). (**g**) Stg, stage. *$P < 0.05$, ***$P < 0.001$ (Student's *t* test).

Supplementary Figure 2. Populations of NK cells and conventional T cells in the liver and spleen in bone marrow-transplanted mice. (**a**) Representative flow cytometry for NK cells ($NK1.1^{+}\beta TCR^{-}$) and conventional T cells ($NK1.1^{-}\beta TCR^{+}$). Numbers of NK cells and conventional T cells in the liver (**b**) and spleen (**c**). Bone marrow cells were isolated from CD45.1 WT mice and transplanted to irradiated CD45.2 WT and CD45.2 LXRα/β-KO mice. After 4 weeks, hepatic MNCs and splenocytes were isolated from recipient WT and LXRα/β-KO mice (n = 6), and analyzed with flow cytometry for iNKT cell populations in CD45.1-gated MNCs. Cell numbers per tissue weight were shown.

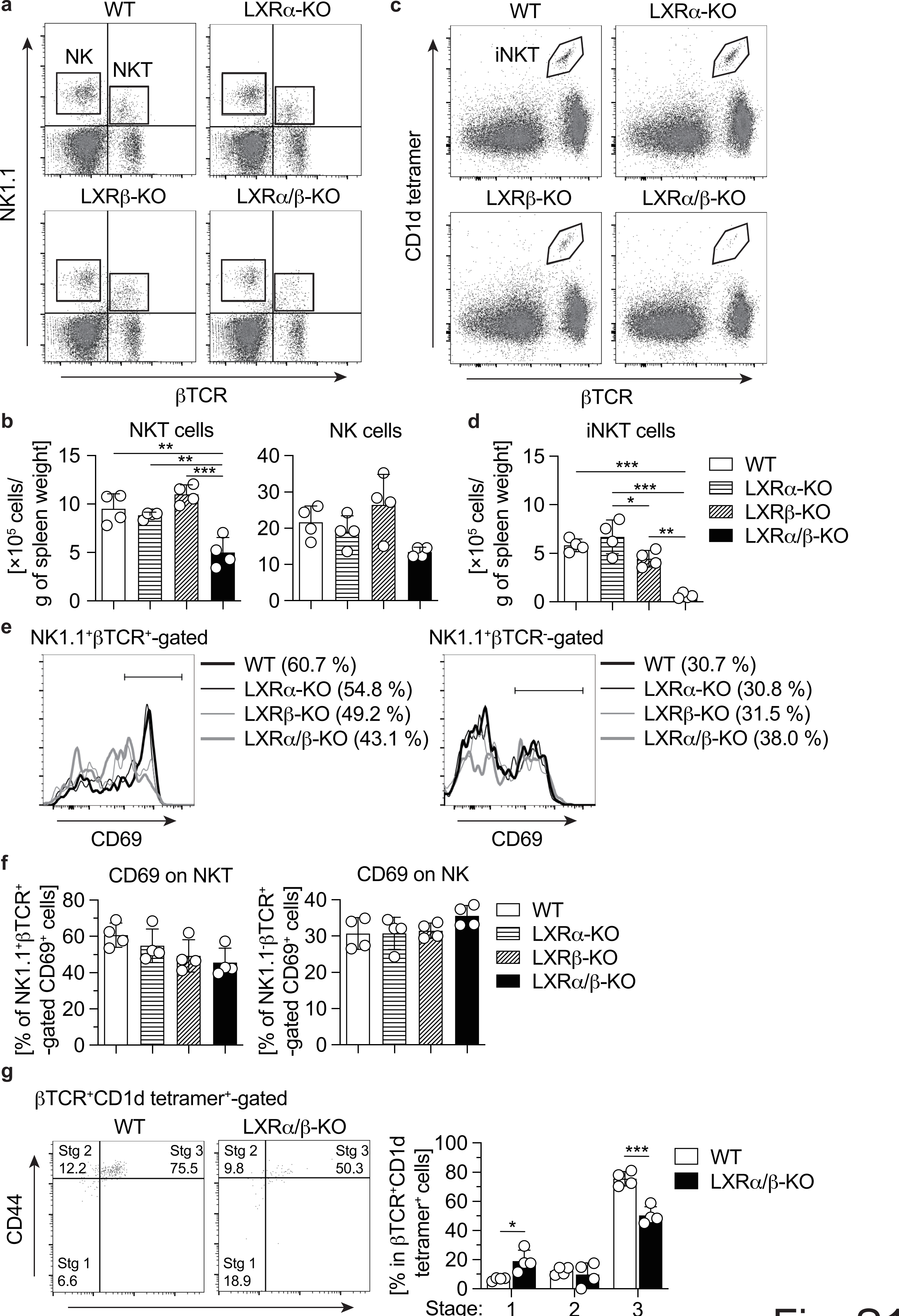
a
WT
LXRα-KO
LXRβ-KO
LXRα/β-KO
NK
NKT
NK1.1
βTCR
c
iNKT
CD1d tetramer
b
NKT cells
NK cells
[×10^5 cells/ g of spleen weight]
d
iNKT cells
e
NK1.1+βTCR+-gated
WT (60.7 %)
LXRα-KO (54.8 %)
LXRβ-KO (49.2 %)
LXRα/β-KO (43.1 %)
NK1.1+βTCR--gated
WT (30.7 %)
LXRα-KO (30.8 %)
LXRβ-KO (31.5 %)
LXRα/β-KO (38.0 %)
CD69
f
CD69 on NKT
CD69 on NK
[% of NK1.1+βTCR+ -gated CD69+ cells]
[% of NK1.1-βTCR+ -gated CD69+ cells]
g
βTCR+CD1d tetramer+-gated
Stg 2 12.2
Stg 3 75.5
Stg 1 6.6
Stg 2 9.8
Stg 3 50.3
Stg 1 18.9
CD44
NK1.1
[% in βTCR+CD1d tetramer+ cells]
Stage:

Fig. S1

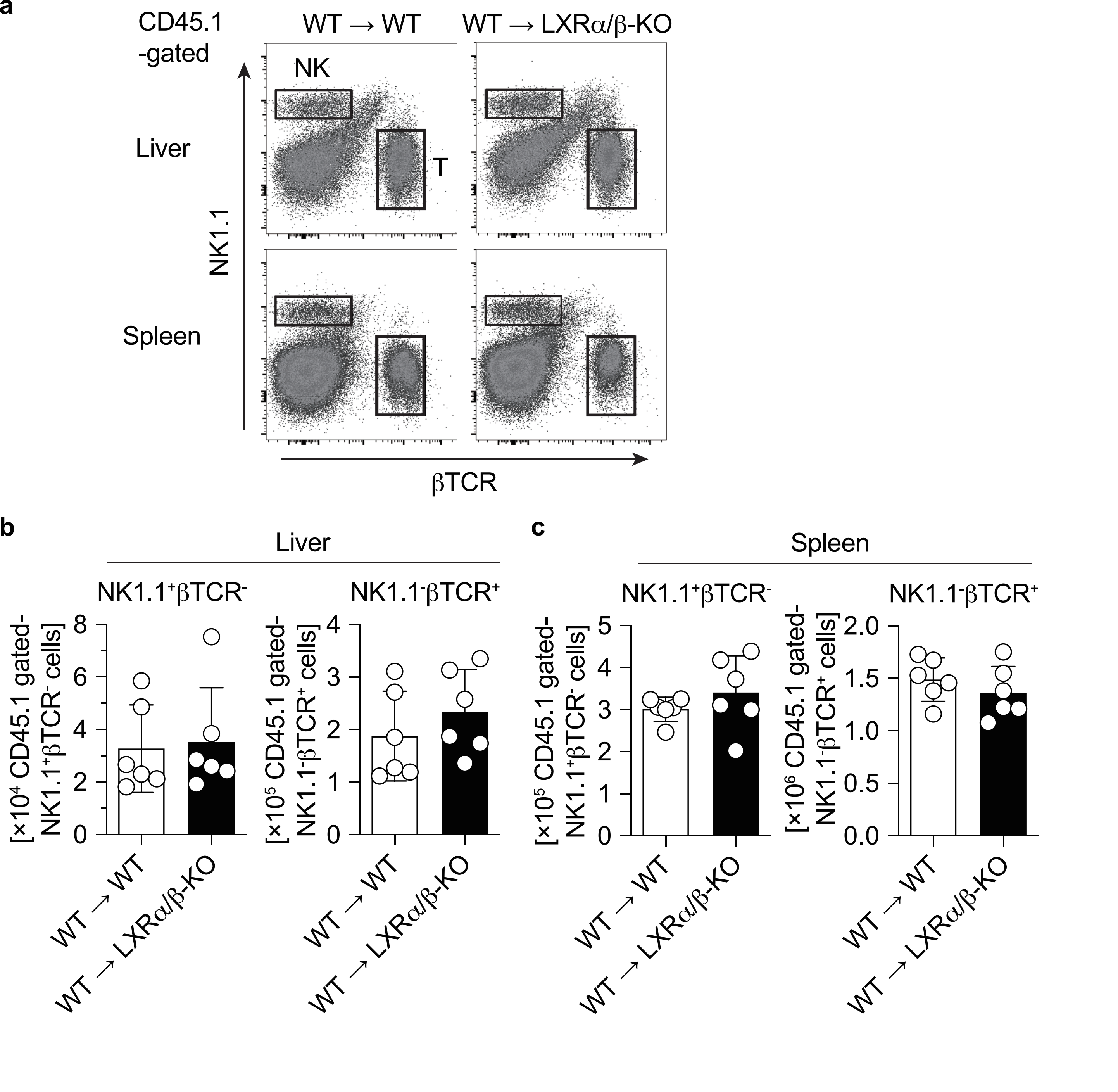

Supplementary Fig. 2
